# Safety Practices in Al-Baha: A Cross-Sectional Study on Parental Awareness of Child Choking Events

**DOI:** 10.7759/cureus.62100

**Published:** 2024-06-10

**Authors:** Khalid Alzahrani, Nahlah A Alzahrani, Sara M Alghamdi, Hamdah A Alshamrani, Haneen A Alghamdi, Mohammad I Barnawi

**Affiliations:** 1 Surgery Department, Faculty of Medicine, Al-Baha University, Al-Baha, SAU; 2 Faculty of Medicine, Al-Baha University, Al-Baha, SAU

**Keywords:** al-baha, saudi arabia, parental knowledge and attitude, foreign body aspiration, child choking

## Abstract

Background: Instances of choking continue to pose a concern for the health and safety of children. This study aims to assess parents' understanding, awareness, and perspectives on child choking.

Methods: A cross-sectional study was conducted in the Al-Baha region of Saudi Arabia from September 13, 2023, to October 3, 2023. Data collection was done via an electronically validated questionnaire among parents aged 18 years and above, covering knowledge, attitudes, and practices. Statistical analysis was performed using the Mann-Whitney U test, the Kruskal-Wallis test, the Shapiro-Wilk test, and the Kolmogorov-Smirnov test. Any result below 0.05 (p < 0.05) was considered significant.

Results: Out of 819 participants, 705 individuals were included in the analysis. The results indicated that there was a good level of knowledge (55%) regarding handling child choking situations. Interestingly, females demonstrated higher levels of awareness compared to males (79.4% versus 20.6%). Attitudes toward managing child choking incidents were rated as overall moderate, with 66.5% showing poor practices, such as being hesitant to seek medical assistance if symptoms improved. A majority of choking cases occurred at home (85%), underscoring the importance of enhancing intervention strategies through increased knowledge dissemination. Notably, the internet and social media platforms (71.8%) emerged as primary sources of information on dealing with child choking incidents. There was significant interest in cardiopulmonary resuscitation (CPR) classes (69.2%), although many people found it hard to make time for them (45%).

Conclusions: Parents in the Al-Baha area seem to have a good understanding but some concerning attitudes when it comes to child choking situations. It is important to spread awareness, correct misconceptions, and encourage CPR training.

## Introduction

Child choking is a difficulty in breathing due to sudden airway obstruction by an object or spasm, a common yet preventable cause of pediatric morbidities and mortalities, with an incidence of around 0.6 cases per 100,000 children [1,2]. Nuts and peanuts are often responsible for choking accidents, with children younger than four years old being more vulnerable [3]. Choking can manifest with varying symptoms, from coughing to severe respiratory failure [4]. Many patients with foreign body aspiration (FBA) may not initially present with signs of choking when they seek help, leading to delayed diagnosis in around 40% of incidents [5].

A detailed history, along with chest X-rays and CT scans, is essential for diagnosing suspected cases of FBA in children [6]. While chest X-rays are good at detecting radiopaque objects stuck in the airways, CT scans provide sensitivity and accuracy for both radiopaque and radiolucent objects [7]. However, bronchoscopy is still considered the method of choice for definitively diagnosing and removing foreign bodies from the airways [8]. The choice of which method to use depends on the child's clinical presentation and risk factors [9,10]. Once FBA is confirmed, it is crucial to remove the object causing the blockage [11]. Early diagnosis of FBA usually reduces damage to the airway lining and lung tissue. On the other hand, delayed diagnosis can increase the risk of complications, such as atelectasis and pneumonia [12,13].

Prevention is a key approach to reducing incidents of FBA. Laws like the Consumer Product Safety Improvement Act of 2008 play a role in this area [14]. Nonetheless, effective prevention also involves educating people about health and raising awareness among parents about the risks of FBA in young children. The American Academy of Pediatrics suggests giving caregivers guidance on preventing choking starting when the child is six months old [15]. Various studies have been conducted both locally in Saudi Arabia and globally regarding public awareness, although the results have been mixed. Given these findings, it is highly recommended to boost public health campaigns to prevent this life-threatening occurrence [16-19]. The extent of knowledge and recognition of choking risks among the general public in Al-Baha, Saudi Arabia, remains underinvestigated. Therefore, the present study was undertaken to assess parents' knowledge, awareness, and attitudes toward child choking in this vital aspect of public health in the region of Al-Baha, Saudi Arabia.

## Materials and methods

A cross-sectional study was conducted in the Al-Baha region of Saudi Arabia from September 13, 2023, to October 3, 2023. The inclusion criteria of our study included adults aged 18 years and above, participants living in the Al-Baha region, and individuals who agreed to participate. On the other hand, the exclusion criteria included the following: those under 18 years old, participants living outside the Al-Baha region, and healthcare professionals (HCPs). Data collection involved an online validated questionnaire in Arabic, shared on social media platforms with the help of data collectors. The validation of the questionnaire was done via a pilot study before data collection. The questionnaire had four parts: demographics, knowledge about child foreign body aspiration, attitudes toward child choking, and sources of knowledge. The questionnaire has been included in the appendices. The study was conducted after obtaining ethical approval from the Institutional Research Board of Al-Baha University (approval number: REC/sur/BU-FM/2023/56R, dated June 12, 2023). The participants were informed about the study aims and assured of data confidentiality, and consent was obtained from each participant before participating in the study.

The results were summarized using statistics such as percentages and means with standard deviations where appropriate. To analyze how participants' socio-demographic factors related to their knowledge, awareness, and attitudes, statistical tests like the Mann-Whitney U test and the Kruskal-Wallis test were used. Any result below 0.05 (p < 0.05) was considered statistically significant. Furthermore, we assessed collinearity using the Shapiro-Wilk and Kolmogorov-Smirnov tests. All statistical analyses were conducted using the IBM SPSS Statistics for Windows, Version 28 (Released 2021; IBM Corp., Armonk, New York) to ensure robust results.

We utilized a scoring system comprising 13 questions to assess participants' knowledge regarding child choking. Each question was coded with a system assigning a value of 1 for correct responses and 0 for incorrect answers or "I don't know" responses. Additionally, Likert scale questions were included, ranging from "disagree" (coded as 1) to "strongly agree" (coded as 5). To lessen bias, negative questions were reverse-coded during scoring. Knowledge scores were calculated by summing up question scores. Participants scoring above the mean were classified as having good knowledge, while those scoring below the mean were categorized as having poor knowledge.

## Results

**Figure 1:**
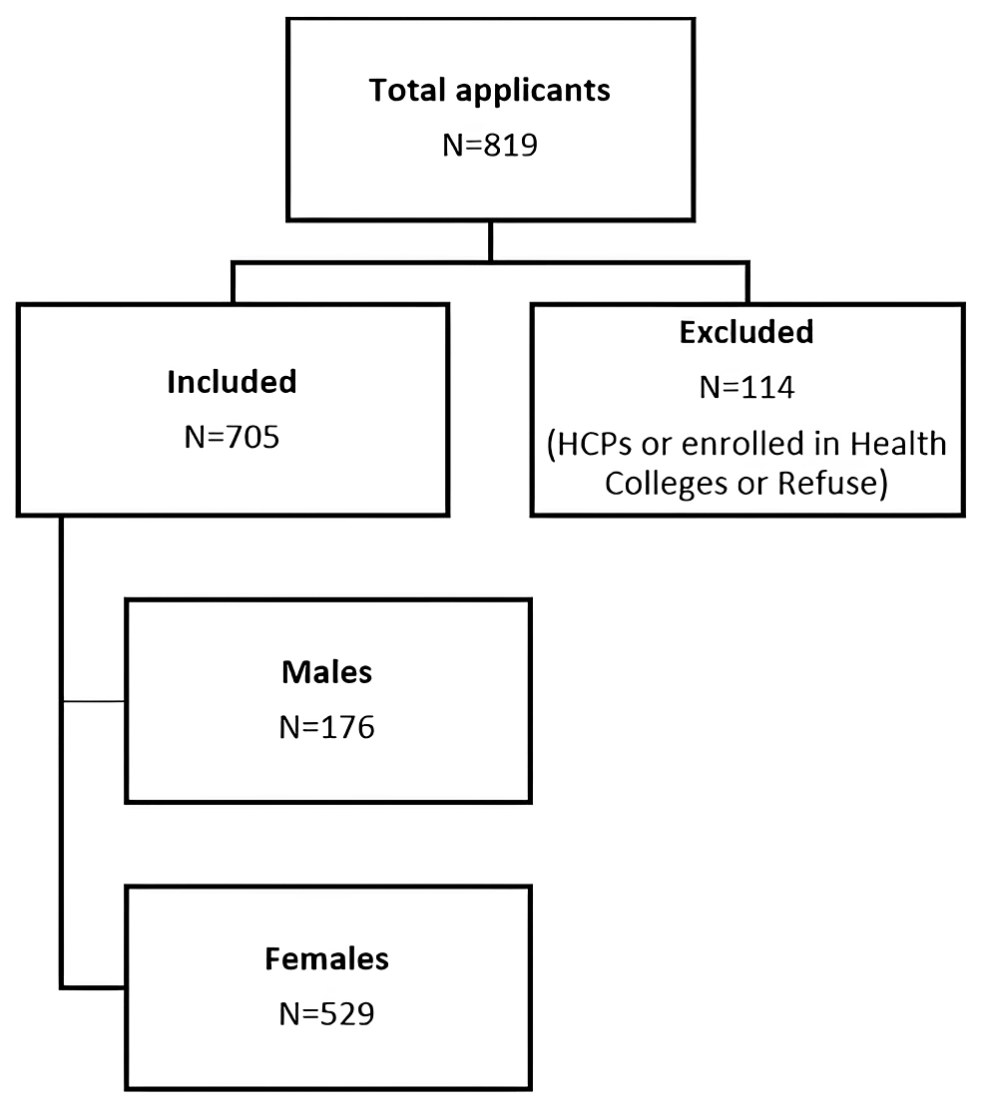
Flow chart for the selection of participants HCPs: healthcare professionals.

A total of 819 individuals applied to participate in the research. After excluding 114 individuals, we had a sample size of 705 participants. The main reasons for exclusion included individuals identified as HCPs and those already enrolled in health colleges, totaling 105 people. Additionally, nine participants dropped out of the study and were therefore not considered (Figure 1).

The study primarily consisted of female participants, making up 75% (n=529) of the sample. A noticeably higher level of knowledge was observed among females (79.4%) compared to their male equivalents (p=0.003). The largest age group was between 41 and 50 years, containing 33.9% of the participants (n=239), with a good level of knowledge within this age group (p=0.43). Most of the participants were married, accounting for 77.4% of the study population (n=546). In terms of education, 66% had completed university-level education (n=465). Employed individuals made up the occupational group at 40.9% (n=288). Regarding family income, the highest proportion fell within the range of 5001-10,000 SR, representing 30.5% of the participants (n=215). There were no significant differences in knowledge levels concerning child choking across these various demographic factors (Tables 1, 2).

**Table 1:** Sociodemographic Characteristics of Study Participants SR: Saudi Riyal.

Sociodemographic Data		n	%
Gender	Male	176	25%
	Female	529	75%
Age	18–30 years	208	29.5%
	31–40 years	169	24%
	41–50 years	239	33.9%
	51–60 years	83	11.8%
	61 years and above	6	0.9%
Marital status	Single	137	19.4%
	Married	546	77.4%
	Divorced or widow	22	3.1%
Educational level	High school or below	180	25.5%
	University	465	66%
	Postgraduate	49	7%
	Uneducated	11	1.6%
Occupation	Employed	288	40.9%
	Unemployed	261	37%
	Retired	62	8.8%
	Student	94	13.3%
Family income	Less than 5000 SR	94	13.3%
	5001–10,000 SR	215	30.5%
	10,001–15,000 SR	180	25.5%
	15,001–20,000 SR	135	19.1%
	More than 20,000 SR	81	11.5%

**Table 2:** Knowledge and Attitude According to Sociodemographic Data ^a^Mann-Whitney test. ^b^Kruskal-Wallis test. SR: Saudi Riyal.

		Knowledge					Attitude				
Sociodemographic data		Poor		Good		P-value	Poor		Good		P-value
		n	%	n	%		n	%	n	%	
Gender^a^	Male	96	54.5%	80	45.5%	0.003	120	68.2%	56	31.8%	0.591
	Female	221	41.8%	308	58.2%		349	66%	180	34%	
Age^b^	18–30 years	81	38.9%	127	61.1%	0.043	121	58.2%	87	41.8%	0.101
	31–40 years	83	49.1%	86	50.9%		125	74%	44	26%	
	41–50 years	102	42.7%	137	57.3%		165	69%	74	31%	
	51–60 years	49	59%	34	41%		55	66.3%	28	33.7%	
	61 years and above	2	33.3%	4	66.7%		3	50%	3	50%	
Marital status^b^	Single	54	39.4%	83	60.6%	0.364	83	60.6%	54	39.4%	0.273
	Married	256	46.9%	290	53.1%		374	68.5%	172	31.5%	
	Divorced or widow	7	31.8%	15	68.2%		12	54.5%	10	45.5%	
Educational level^b^	High school or below	78	43.3%	102	56.7%	0.356	116	64.4%	64	35.6%	0.730
	University	208	44.7%	257	55.3%		315	67.7%	150	32.3%	
	Postgraduate	23	46.9%	26	53.1%		30	61.2%	19	38.8%	
	Uneducated	8	72.7%	3	27.3%		8	72.7%	3	27.3%	
Occupation^b^	Employed	135	46.9%	153	53.1%	0.224	191	66.3%	97	33.7%	0.737
	Unemployed	117	44.8%	144	55.2%		178	68.2%	83	31.8%	
	Retired	32	51.6%	30	48.4%		42	67.7%	20	32.3%	
	Student	33	35.1%	61	64.9%		58	61.7%	36	38.3%	
Family income^b^	<5000 SR	43	45.7%	51	54.3%	0.690	64	68.1%	30	31.9%	0.897
	5001–10,000 SR	93	43.3%	122	56.7%		141	65.6%	74	34.4%	
	10,001–15,000 SR	82	45.6%	98	54.4%		121	67.2%	59	32.8%	
	15,001–20,000 SR	60	44.4%	75	55.6%		90	66.7%	45	33.3%	
	>20,000 SR	39	48.1%	42	51.9%		53	65.4%	28	34.6%	

**Figure 2:**
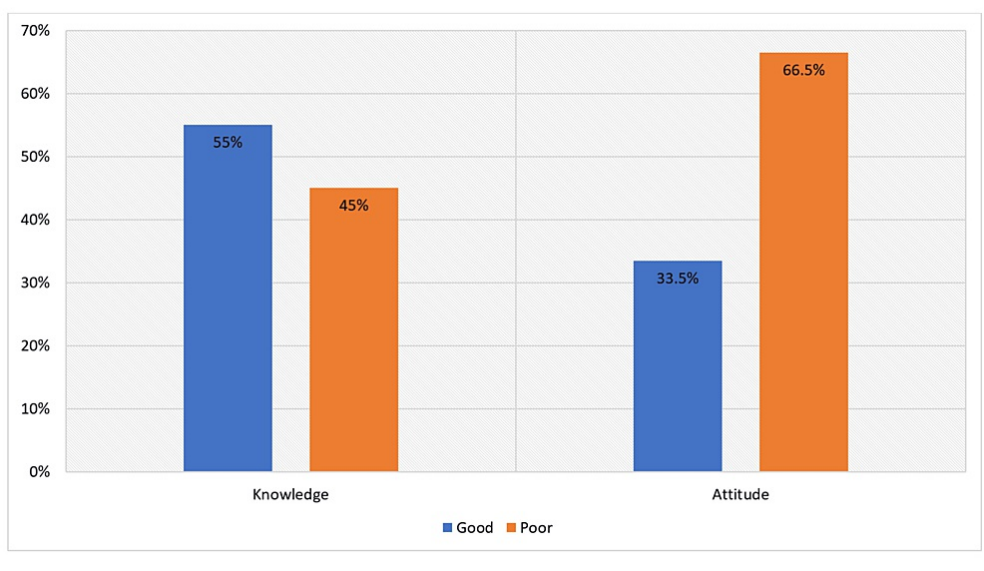
Level of Knowledge and Attitude

In our analysis of parents' understanding and awareness of child choking and how to prevent it, we discovered that 55.0% of parents demonstrated a good level of knowledge (Figure 2), with an average knowledge score of 20.3 out of 25, indicating a moderate level of knowledge (Table 3).

**Table 3:** Assessment of Level of Knowledge *Indicates correct answer. ^†^Variable with multiple response answers.

Question	Answers	n	%
1. I have sufficient knowledge about choking in children	Strongly disagree	21	3%
	Disagree	97	13.8%
	Neutral	197	27.9%
	Agree	304	43.1%
	Strongly agree*	86	12.2%
2. At what age are children most at risk of choking?	Less than 1 year	208	29.5%
	1–5 years*	462	65.5%
	6–10 years	32	4.5%
	More than 10 years	3	0.4%
3. What are the materials most likely to cause choking in children?^†^	Coins*	500	70.9%
	Small toys*	473	67.1%
	Nuts*	416	59%
	Liquids	106	15%
	Dessert*	295	41.8%
	Batteries*	337	47.8%
	Any small pieces*	592	84%
4. Removing the above materials reduces the risk of suffocation.	No	28	4%
	Yes*	644	91.3%
	I don't know	33	4.7%
5. Nuts should not be given to children under 5 years old.	No	77	10.9%
	Yes*	588	83.4%
	I don't know	40	5.7%
6. Does talking and laughing while eating cause choking?	No	27	3.8%
	Yes*	650	92.2%
	I don't know	28	4%
7. What are the signs of choking?^†^	Cough*	412	58.4%
	Apnea*	637	90.4%
	Abdominal pain	17	2.4%
	Facial color change*	611	86.7%
	Inability to speak*	546	77.4%
	Fever	12	1.7%
	Holds his/her neck or waves his/her hand*	548	77.7%
	Loss of consciousness*	334	47.4%
8. What are the possible complications of choking?^†^	Brain death*	273	38.7%
	Death*	511	72.5%
	Airway injury*	570	80.9%
	Cardiac arrest*	325	46.1%
	Stroke	107	15.2%
	There are no complications	13	1.8%
9. How can choking in children be avoided?^†^	Remove potential items (coins, nuts, small toys, batteries, etc.)*	663	94%
	Supervising children’s playing and eating*	585	83%
	Prevent children from talking and laughing while eating*	475	67.4%
	Divide food into small portions*	454	64.4%
	Drink water while eating	262	37.2%
	I don't know	14	2%

As for parents' attitudes toward child choking and its prevention, it was noted that a significant percentage (66.5%) showed a poor attitude toward this issue (Figure 2). The average attitude score was calculated to be 4.1 out of 7. In our study, we found that 55.3% (n=390) would not seek medical care in case of symptom relief (Table 4).

**Table 4:** Assessment of Attitude *Indicates correct answer.

Question	Answers	n	%
1. Do you let your child eat without supervision?	Always	19	2.7%
	Sometimes	320	45.4%
	Rarely	197	27.9%
	Never*	169	24%
2 A. If you witnessed a child choking incident now, what would you do?	Did not do anything	14	2%
	Remove the cause of choking from the child's mouth with your fingers*	130	18.4%
	Tried hitting the back and pressing the stomach when the child was choking	421	59.7%
	Ask for help from the people around you	78	11.1%
	Contact the Red Crescent	62	8.8%
2 B. If the answer to the previous question is (I will not do anything), what is the reason?	Insufficient experience	5	35.7%
	Fear of legal penalties	1	7.1%
	Fear of harming the victim	8	57.1%
3. Do you take the child to the hospital when he or she suffocates?	No	83	11.8%
	Yes*	542	76.9%
	I don't know	80	11.3%
4. If the symptoms stop, do you go to the hospital?	No	390	55.3%
	Yes*	261	37%
	I don't know	54	7.7%

Our research indicated that among the participants, 57.7% had witnessed incidents of child suffocation, with a high percentage (84.5%) involving family members. Approximately half of the participants (52.3%) tried to help by patting the child's back and applying pressure on the stomach (Table 5).

**Table 5:** Witnessing Child Suffocation Incidents

Question	Answers	n	%
1. If you have ever witnessed a child suffocate, what is the relationship between you and him/her?	From family members	344	84.5%
	A member of a friend's family	32	7.9%
	Stranger	31	7.6%
2. If you have ever witnessed a child choking incident, where did the incident occur?	Public place	50	12.3%
	The school	11	2.7%
	The home	346	85.0%
3. If you ever witnessed a child choking incident, how did you react to it?	Did not do anything	31	7.6%
	Remove the cause of choking from the child's mouth with your fingers	86	21.1%
	Tried hitting the back and pressing the stomach when the child was choking	213	52.3%
	Ask for help from the people around you	67	16.5%
	Contact the Red Crescent	10	2.5%

Upon analyzing our data, we found that the majority (71.8%) relied on the Internet and social media as their sources of information. In terms of awareness regarding CPR courses, 58% were not familiar with such courses in the Al-Baha region; however, a considerable portion (69.2%) expressed interest in enrolling in these courses. The primary factor cited for not signing up for CPR classes was a lack of time, accounting for 45.0% of the respondents (Table 6).

**Table 6:** Exploring Information Sources and Learning Preferences CPR: cardiopulmonary resuscitation.

Question	Answers	n	%
1. Source of your information?	Internet and social media	495	71.8%
	Family and friends	445	64.6%
	Health practitioners	228	33.1%
	Attending first aid courses	184	26.7%
	Experience gained from the workplace	12	1.7%
2. What method do you prefer to learn the correct procedures?	Awareness clips	626	89.3%
	Text messages	150	21.4%
	Representative pictures	413	58.9%
	Self-reading	215	30.7%
3. Have you heard about CPR courses in the Al-Baha region?	No	409	58%
	Yes	296	42%
4. Do you have an interest in enrolling in CPR courses?	No	217	30.8%
	Yes	488	69.2%
5. If you have not taken CPR courses before, why not?	I do not care	62	8.8%
	Shortage of time	317	45%
	I had never heard of a CPR course before	269	38.2%
	Financial cost	42	6%
	The place is very far	2	0.3%
	I attended it beforehand	12	1.7%

## Discussion

Child choking poses a significant risk leading to both child mortality and morbidity [1]. The level of awareness among parents is a contributing factor to this ongoing problem. Therefore, it is crucial to involve parents by increasing their knowledge to reduce the dangers associated with choking incidents for children [2]. According to our research, 55% of parents displayed a good level of knowledge, scoring an average of 20.3 out of 25 points. This finding is consistent with Shakhs et al. in the Eastern region of Saudi Arabia, where 60.3% of participants showed awareness of airway obstruction [20]. Another study in Turkey highlighted mothers' strong knowledge of preventing incidents [16]. However, several studies point out parental unawareness regarding child choking risks [21,22]. Interestingly, higher levels of awareness were observed among parents in the Al-Baha region (57.7%, n=407), possibly due to previous experiences with choking incidents and increased exposure to social media content over time.

In our study, we discovered that certain parental characteristics, particularly female gender, were linked to a higher level of knowledge (79.4%, p=0.003). This finding aligns with existing literature [22,23]. Our result could be influenced by factors such as societal roles, education, and caregiving duties often associated with women in various societies. Moreover, there is a possibility of self-selection bias due to the female participant composition, implying that the knowledge level of male participants may not be accurately reflected in the data.

A concerning aspect highlighted in our study is that 66% of participants demonstrated a poor attitude, evident from an average score of 4.1 out of 7. The alarming fact that 55% of parents wouldn't seek help if symptoms improve points towards a significant issue requiring attention and effective solutions. Timely diagnosis in cases like FBA is crucial to prevent complications; therefore, addressing this issue promptly is essential [12,13]. The decision to avoid seeking assistance despite symptom relief could arise from various reasons such as misconceptions about the seriousness of the situation or limited understanding of potential long-term risks and complications. Based on our research, 2% of respondents hesitated to help during a child's choking incident due to fear of causing harm or lack of experience. 

We propose several solutions, including launching public awareness campaigns to debunk myths and equip people with first aid skills, promoting a culture of assistance by showcasing successful interventions, and emphasizing the importance of taking action.

The key to managing FBA lies in prevention rather than treatment [24]. Our study showed that participants demonstrated understanding regarding preventive measures. Most people agreed that children below the age of five should not be given nuts, and they recognized the risks associated with talking or laughing during meals due to the risk of choking. Additionally, most participants recognized that coins are a major cause of choking in children. As a result, they preferred to keep small items out of their child's reach as a preventive measure. This proactive approach to limiting access to choking hazards is an important step in preventing incidents of FBA in children [25].

The information sources on FBA in our study are quite diverse, with 33.1% coming from health practitioners. This highlights the need for health authorities to increase awareness and share information. The prevalence of misinformation in media underscores the importance of reliable sources. Additionally, 26.7% of participants gained knowledge from first aid courses, which are considered trustworthy sources. Interestingly, many participants showed interest in CPR courses. Obstacles like time constraints and lack of information about course availability delayed their participation. Therefore, it is essential to develop strategies to promote these courses effectively and make them more accessible and well-known.

Our study has several limitations. Firstly, the cross-sectional design does not account for time-cause relationships, which could impact how results are interpreted. Another point to consider is the bias stemming from self-reported data. Additionally, focusing on one area in Saudi Arabia could make it difficult to apply the findings to broader populations and different locations. It's important to keep these constraints in mind when analyzing results and planning future studies.

## Conclusions

Parents in the Al-Baha region showed a good level of knowledge regarding FBA and an acceptable understanding of how to prevent choking incidents. However, they seemed hesitant about dealing with cases of FBA, particularly when it comes to seeking medical care in case of symptom improvement after an FBA incident. They mainly rely on the internet and social media for information, while healthcare professionals play a minor role in information dissemination. Addressing these issues is crucial to ensure that parents are adequately prepared to manage FBA incidents and seek timely medical care.

## Appendices

The data collection questionnaire used in this study is presented in Table 7.

**Table 7:** Data collection questionnaire CPR: cardiopulmonary resuscitation.

Questionnaire
Safety Practices in Albaha: Cross-Sectional Study on Parental Awareness of Child Choking Events
We are a research group from Al-Baha University aiming to assess knowledge, awareness, and attitude toward child choking among parents
Do you agree to participate in the study?
Yes
No
Do you work as a healthcare professional?
Yes
No
Are you living in Al-Baha?
Yes
No
Number of data collector:
………
Section A (demographic data)
Gender:
Male
Female
Age:
18–30 years
31–40 years
41–50 years
51–60 years
61 years and above
Marital status:
Single
Married
Divorced or widow
Educational level:
High school or below
University
Post-graduate
Uneducated
Occupation:
Employed
Unemployed
Retired
Student
Family income:
Less than 5,000 SR
5,001–10,000 SR
10,001–15,000 SR
15,001–20,000 SR
More than 20,000 SR
Section B: Assessment of the level of knowledge:
1. I have sufficient knowledge about choking in children?
Strongly disagree
Disagree
Neutral
Agree
Strongly agree
2. At what age are children most at risk of choking?
Less than 1 year
1–5 years
6–10 years
More than 10 years
3. What are the materials most likely to cause choking in children?
Coins
Small toys
Nuts
Liquids
Dessert
Batteries
Any small pieces
4. Is removing the above materials reduce the risk of suffocation?
No
Yes
I don't know
5. Are nuts should not be given to children under 5 years old?
No
Yes
I don't know
6. Does talking and laughing while eating cause choking?
No
Yes
I don't know
7. What are the signs of choking?
Cough
Apnea
Abdominal pain
Facial color change
Inability to speak
Fever
Holds his/her neck or waves his/her hand
Loss of consciousness
8. What are the possible complications of choking?
Brain death
Death
Airway injury
Cardiac arrest
Stroke
There are no complications
9. How can choking in children be avoided?
Remove potential items (coins, nuts, small toys, batteries, etc.)
Supervising children’s playing and eating
Prevent children from talking and laughing while eating
Divide food into small portions
Drink water while eating
I don't know
Section C: Assessment of Attitude
10. Do you let your child eat without supervision?
Always
Sometimes
Rarely
Never
11A. If you witnessed a child choking incident now, what would you do?
Did not do anything
Remove the cause of choking from the child's mouth with your fingers
tried hitting the back and pressing the stomach when the child is choking
Ask for help from the people around you
Contact the Red Crescent
11B. If the answer to the previous question is (I will not do anything), what is the reason?
Insufficient experience
Fear of legal penalties
Fear of harming the victim
12. Do you take the child to the hospital when he or she suffocates?
No
Yes
I don't know
13. If the symptoms stop, do you go to the hospital?
No
Yes
I don't know
14. If you have ever witnessed a child suffocate, what is the relationship between you and him/her?
From family members
A member of a friend's family
Stranger
15. If you have ever witnessed a child choking incident, where did the incident occur?
Public place
The school
The home
16. If you ever witnessed a child choking incident, how did you react to it?
Did not do anything
Remove the cause of choking from the child's mouth with your fingers
Tried hitting the back and pressing the stomach when the child was choking
Ask for help from the people around you
Contact the Red Crescent
Section D: Assessment of source of knowledge
17. Source of your information?
Internet and social media
Family and friends
Health practitioners
Attending first aid courses
Experience gained from the workplace
18. What method do you prefer to learn the correct procedures?
Awareness clips
Text messages
Representative pictures
Self-reading
19. Have you heard about CPR courses in Al-Baha region?
No
Yes
20. Do you have an interest in enrolling in CPR courses?
No
Yes
21. If you have not taken CPR courses before, why not?
I do not care
Shortage of time
I had never heard of a CPR course before
Financial cost
The place is very far
I attended it beforehand
